# Cleavage and Polyadenylation Specific Factor 1 Promotes Tumor Progression *via* Alternative Polyadenylation and Splicing in Hepatocellular Carcinoma

**DOI:** 10.3389/fcell.2021.616835

**Published:** 2021-03-04

**Authors:** Shi-lu Chen, Zhong-xu Zhu, Xia Yang, Li-li Liu, Yang-fan He, Ming-ming Yang, Xin-yuan Guan, Xin Wang, Jing-ping Yun

**Affiliations:** ^1^State Key Laboratory of Oncology in South China, Collaborative Innovation Center for Cancer Medicine, Sun Yat-sen University Cancer Center, Guangzhou, China; ^2^Department of Pathology, Sun Yat-sen University Cancer Center, Guangzhou, China; ^3^Department of Biomedical Sciences, City University of Hong Kong, Hong Kong, China; ^4^Key Laboratory of Biochip Technology, Biotech and Health Centre, Shenzhen Research Institute, City University of Hong Kong, Shenzhen, China

**Keywords:** hepatocellular carcinoma, alternative polyadenylation, cleavage and polyadenylation specific factor 1, cleavage and polyadenylation, alternative splicing

## Abstract

Alternative polyadenylation (APA) is an important post-transcriptional regulatory mechanism required for cleavage and polyadenylation (CPA) of the 3′ untranslated region (3′ UTR) of mRNAs. Several aberrant APA events have been reported in hepatocellular carcinoma (HCC). However, the regulatory mechanisms underlying APA remain unclear. In this study, we found that the expression of cleavage and polyadenylation specific factor 1 (CPSF1), a major component of the CPA complex, was significantly increased in HCC tissues and correlated with unfavorable survival outcomes. Knockdown of CPSF1 inhibited HCC cell proliferation and migration, whereas overexpression of CPSF1 caused the opposite effect. Based on integrative analysis of Iso-Seq and RNA-seq data from HepG2.2.15 cells, we identified a series of transcripts with differential 3′ UTR lengths following the knockdown of CPSF1. These transcripts were related to the biological functions of gene transcription, cytoskeleton maintenance, and endomembrane system transportation. Moreover, knockdown of CPSF1 induced an increase in alternative splicing (AS) events in addition to APA. Taken together, this study provides new insights into our understanding of the post-transcriptional regulatory mechanisms in HCC and implies that CPSF1 may be a potential prognostic biomarker and therapeutic target for HCC.

## Introduction

Liver cancer is one of the most aggressive malignancies worldwide, with 841,080 new cases and 781,631 cancer-related deaths reported in 2018 (Bray et al., 2018). According to GLOBOCAN statistics, the incidence and mortality rate of liver cancer were 905,677 and 830,180, respectively, in 2020. Hepatocellular carcinoma (HCC) constitutes approximately 80% of primary liver cancer cases and has extremely poor prognosis. Recent studies have identified many liver-specific oncogenes and tumor suppressor genes that control HCC growth and metastasis, that have been driving research in targeted therapies (Chen et al., 2020). However, the improvement in HCC survival rate is still not acceptable; HCC ranked fourth as a leading cause of cancer-related deaths in 2018 because of poor response to systemic therapies. Therefore, it is necessary to continue searching for potential prognostic and therapeutic targets for HCC through enhancing our understanding of its cellular biological processes.

Post-transcriptional regulation is a common and vital regulatory mechanism that contributes to the diversity of gene expression. Owing to the rapid development of next-generation RNA sequencing, various post-transcriptional alterations in RNA processing have been discovered. In eukaryotic cells, the maturation of primary messenger RNAs (pre-mRNAs) involves 5′ untranslated region (5′ UTR) modification, removal of introns by splicing, and 3′ untranslated region (3′ UTR) cleavage and polyadenylation (CPA). Alternative polyadenylation (APA) is a general and evolutionarily conserved mechanism of transcriptional modification that involves endonucleolytic cleavage and addition of a polyadenosine tail of 200–250 nucleotides that generates different lengths of the 3′UTR from a single gene (Wang et al., 2008; Di Giammartino et al., 2011). APA at the RNA 3′UTR end of RNA results in various transcript isoforms that contribute to transcriptome diversity. Instead of affecting the protein-coding region, the 3′ UTR region contains many recognition elements for microRNAs and RNA-binding proteins (RBPs) that affect the subcellular localization, intracellular trafficking, degradation, and translation rate of the transcripts in different cellular contexts (Mayr, 2016). Recent studies have revealed that APA is linked to changes in cellular states, including cancer and other diseases (Gruber and Zavolan, 2019). In HCC, a large number of APA events had been identified (Xiang et al., 2018). However, only a few studies have investigated the underlying mechanisms of APA and tumor progression. Thus, it is essential to identify the factors that control APA in HCC.

Alternative polyadenylation is mediated by a group of constant proteins that constitute the CPA multicomponent complexes, including four core components: cleavage and polyadenylation specific factors (CPSFs), cleavage stimulation factors (CSTFs), cleavage factor I (CFI), and CFII, along with numerous dynamic partners (Tian and Manley, 2017). CPSFs are site-specific cleavage subunits composed of CPSF1, CPSF2, CPSF3, CPSF4, and Fip1. As the largest component of the CPSFs complex, CPSF1 (also known as CPSF160) is primarily located in the nucleus and functions in pre-mRNA 3′UTR processing by recognizing a conserved AAUAAA polyadenylation signal near the polyadenylation site (pA site) (Murthy and Manley, 1995). Differential expression of CPSF1 has been reported to be closely associated with the progression of multiple human diseases. For example, CPSF1 binds to influenza virus NS1, resulting in the inhibition of wild-type influenza A virus replication (Du et al., 2020). CPSF1 expression decreases in diabetic retinopathy and mediates retinal vascular dysfunction *via* the MAPK/ERK pathway (Zhang et al., 2020). CPSF1 mutations are associated with early onset high myopia and involved in retinal ganglion cell axon projection (Ouyang et al., 2019). In tumors, CPSF1 plays an oncogenic role in head and neck squamous cell carcinoma (Sakai et al., 2020), ovarian cancer (Zhang et al., 2017), and prostate cancer growth (Van Etten et al., 2017). However, its potential role in HCC has not been fully investigated.

In the present study, we investigated the clinical significance and biological function of CPSF1 in HCC. We found that CPSF1 was significantly upregulated in HCC. As a critical regulator of APA in HCC, CPSF1 accelerated cell proliferation and was correlated with unfavorable outcomes in patients with HCC. Our findings describe the oncogenic role of CPSF1 in HCC and suggest its potential role as a novel prognostic biomarker for HCC.

## Materials and Methods

### Patients, Tissue Specimens, and Follow-Up

A total of 796 patients with primary HCC who underwent surgery between January 2000 and December 2010 at the Sun Yat-sen University Cancer Center (SYSUCC, Guangzhou, China) were included in this study. Paraffin-embedded tissue samples from these patients were re-embedded in new paraffin blocks for tissue microarrays (TMAs). Normal tissues were obtained from no less than 1 cm adjacent to tumor margin. This study was approved by the Institute Research Medical Ethics Committee of SYSUCC. All patients provided written informed consent for the use their tissues and data for research purposes. Pathological specimens and data were collected, and all samples were rendered anonymous. The overall survival (OS) follow-up period was defined as the interval from the date of surgery to the date of death or last follow-up. None of the patients received radiotherapy or chemotherapy prior to surgery.

### Hematoxylin–Eosin and Immunohistochemistry Staining

The HCC TMA blocks were cut into 4-μm slices and mounted onto glass slides. These slides were then dewaxed and treated with 3% hydrogen peroxide in methanol and blocked with a Biotin-Blocking Kit (DAKO, Glostrup, Germany). HE and IHC staining of CPSF1 (1:200, Bethyl, A301-580) were then performed, and the slides were assessed by two independent pathologists who calculated the respective IHC scores. The IHC scores were determined by multiplying the staining intensity by the proportion of stained nuclei. The staining intensity was scored as four grades (0, 1, 2, and 3), which indicated a staining intensity from blank to strong staining. The proportion was scored as five grades (0, 1, 2, 3, and 4), which indicated a staining area of 0, 1–25, 25–50, 50–75, and 75–100%, respectively. The median IHC score was used as the cutoff value to separate patients into high and low CPSF1 expression groups.

### Cell Culture

Liver cell line QSG7701, and HCC cell lines HepG2.2.15 and HCCLM3 were obtained from the Cell Bank of Type Culture Collection of Chinese Academy of Sciences Committee (Shanghai, China), and cultured in Dulbecco’s modified Eagle’s medium (Gibco, Grand Island, NY, United States) supplemented with 10% fetal bovine serum (Gibco, South America). All cells were grown in a humidified atmosphere at 37°C with 5% CO_2_.

### Generation of Cells With CPSF1 Overexpression and Knockdown

Full-length CPSF1 cDNA was cloned into the mammalian vector pENTER for overexpression and confirmed by sequencing. The plasmid was transfected into HCC cell lines using Lipofectamine^TM^ 3000 reagent (Invitrogen, Carlsbad, CA, United States). For knockdown assays, small interfering RNAs (siRNAs) targeting CPSF1 were purchased from Shanghai GenePharma Co., Ltd. (Shanghai, China). Transfection was performed using Lipofectamine^TM^ RNAiMAX (Invitrogen). The siRNA sequences used are listed in Supplementary Table 1.

### Protein Extraction and Western Blot Analysis

Total cellular proteins were extracted and resuspended in lysis buffer (Beyotime Biotechnology, Shanghai, China) supplemented with a protease inhibitor. Western blotting was performed using a standard protocol described previously (Chen et al., 2019). Briefly, total proteins (30 μg) were loaded in individual lanes and separated on an 8% sodium dodecyl sulfate-polyacrylamide gel electrophoresis (SDS-PAGE) gel. The following primary antibodies against the indicated proteins were used: CPSF1 (1:1,000, Bethyl, A301-580) and β-actin (1:2,000, Santa Cruz Biotechnology, Dallas, TX, United States). The secondary antibodies used were anti-rabbit (Cell Signaling Technology, 7074S, 1:3,000) and anti-mouse (Cell Signaling Technology, 7076S, 1:3,000), and both were purchased from Cell Signaling Technology. The protein band intensities were quantified using the Bio-Rad Molecular Imager ChemiDoc^TM^ XRS + system.

### RNA Extraction and Quantitative Real-Time RT-PCR

Total RNA was isolated from fresh tissues and cell lines, and purified using TRIzol reagent (BIOO Scientific Co., Austin, TX, United States) according to the manufacturer’s instructions. The mRNA was reverse-transcribed into cDNA using standard procedures with a Reverse Transcriptase Kit (Vazyme Biotech, Nanjing, China). SYBR Green-based quantitative real-time PCR (qRT-PCR) (Vazyme Biotech) was performed in 96-well plates. The mRNA levels were normalized to the expression of 18S mRNA. For APA validation, we used percentage of distal polyadenylation site usage index (PDUI) value by analyzing PDUI = long 3′UTR transcript/total transcript. For AS validation, we used percent spliced-in (PSI) value by analyzing PSI = skipping exon (SE) transcript/total transcript. Sequences of the oligonucleotide primers used for each transcript are listed in Supplementary Table 1.

### PacBio Sequencing and Transcriptome Construction by PacBio Reads

According to the Iso-Seq-v3 analysis pipeline^1^, subreads were used to call circular consensus sequences. Polished full-length non-concatemer (FLNC) reads were generated after primer, concatemer removal, and clustering. The FLNC reads were aligned against the reference genome (hg19/GRCh37) using GMAP ALIGNER (v2019-03-15) (Wu and Watanabe, 2005). Based on genome alignment, isoforms were collapsed by cDNA_Cupcake^2^, followed by comparison with reference annotation (RefSeq hg19) using Cuffcompare (Trapnell et al., 2010). Isoforms with a complete match of the intron chain and potentially novel isoforms (with at least one known splice junction and occurring in both libraries) were selected. We then constructed a full transcriptome. The constructed transcriptome was used to identify alternative splicing (AS) events by SUPPA2 (Trincado et al., 2018). Seven types of AS events were identified, including SE, mutually exclusive exons (MX), alternative 5′ splicing site (A5), alternative 3′ splicing site (A3), retained intron (RI), alternative first exon (AF), and alternative last exon (AL).

### RNA-seq Data and Enrichment Analyses

Total RNA was extracted from HepG2.2.15 cells transfected with CPSF1 or scrambled siRNAs. Libraries were prepared using TruSeq Stranded mRNA Kits (Illumina) and subjected to 150 cycle paired-end sequencing on the Illumina HiSeq platform. Each group was sequenced in three replicates. Quality control and preprocessing of the sequencing data were performed using fastp-0.20.1 (Chen S. et al., 2018). Based on the constructed transcriptome, the abundance of transcripts was quantified by Salmon-1.0.0 (Patro et al., 2017) and gene models were generated in BED format. Wiggle files indicative of read coverage were generated by GenomeCoverageBed (BEDTools, v2.26.0) after sequence alignment by STAR-2.7.0f (Quinlan and Hall, 2010). Then, APA event analysis was performed using DaPars (version 0.9.1) to predict proximal polyadenylation sites and distal polyadenylation sites (Masamha et al., 2014). Significant APA events were determined using three criteria. First, the *P*-value of the PDUI differences should be <0.05. Second, the absolute mean difference of the PDUI must be ≥0.2. Third, the mean PDUI fold change must be ≥1.5. For functional analysis of the transcripts, Kyoto Encyclopedia of Genes and Genomes (KEGG) and Gene Ontology (GO) gene sets were analyzed using DAVID^3^. For motif analysis, sequences between proximal and distal polyadenylation sites were collected in FASTA format and submitted to MEME suits^4^. For network analysis of functional protein association, CPSF1-associated proteins were filtered using experimental or curated data from the STRING database^5^. For APA data analyzed by DaPars, we downloaded the PDUI values of transcripts from^6^.

### Migration and Invasion Assays

Following transfection, 5 × 10^4^ cells were harvested and replated in the upper compartment of Transwell chambers (8-μm pore size, Millipore, Billerica, MA, United States) in serum-free medium. For invasion assay, the upper chambers were coated with 50 μL of 0.5 mg/mL Matrigel^®^. Fresh medium containing 10% fetal bovine serum was placed in the lower chamber. The fetal bovine serum-containing medium in the lower chamber serves as a chemoattractant for cell migration. After incubation for 36 h, the cells in the lower membrane were fixed with methanol for 30 min and then stained with 0.1% crystal violet. Three 20× magnification fields were randomly chosen to count the cell number under a microscope.

### CCK8 and Colony Formation Assays

For Cell Counting Kit-8 (CCK8) assays, HCC cells were plated in 96-well plates and pretreated by transfection with CPSF1 plasmids or siRNAs. The cells were quantified on five consecutive days by incubating with the CCK8 detection reagent for 2 h at 37°C followed by colorimetric reading of absorbance at 450 nm using a microplate reader. For the colony formation assay, single-cell suspensions were plated in six-well plates. The culture medium was replaced every 72 h. After 14 days, the cells were fixed in methanol for 10 min, stained with crystal violet, and washed twice in PBS. Images were captured using a digital scanner to count the colonies.

### Anchorage-Independent Growth

Cultured HCC cells were plated in six-well plates and transfected with CPSF1 plasmids or siRNAs. Equal number of cells (1,000 cells) from each group was embedded in 2 mL of 0.35% agar solution and layered on top of 1.5 mL of 0.6% basal agar layer pre-coated in a six-well plate. Fresh medium (200 μL) was added every 3 days. The plates were incubated at 37°C with 5% CO_2_ for 3 weeks. Colony formation was determined by counting colonies larger than 50 μm in 10 fields under the 4× magnification under a microscope.

### Statistical Analyses

All data are presented as the mean ± standard deviation. Statistical analyses were performed using SPSS (Version 20.0, IBM, Armonk, NY, United States), R (Version 3.6), and GraphPad PRISM software (Version 7.0, GraphPad Software Inc., La Jolla, CA, United States). Student’s *t*-test, Pearson’s χ^2^ test, Kruskal–Wallis analysis, ANOVA analysis, Fisher’s exact test, Kaplan–Meier method, and multivariate Cox proportional hazard regression models were used to analyze independent prognostic factors in OS and disease-free survival (DFS). *P* < 0.05 (two-tailed) was considered statistically significant.

## Results

### CPSF1 Is Highly Expressed in HCC Tissues

**FIGURE 1:**
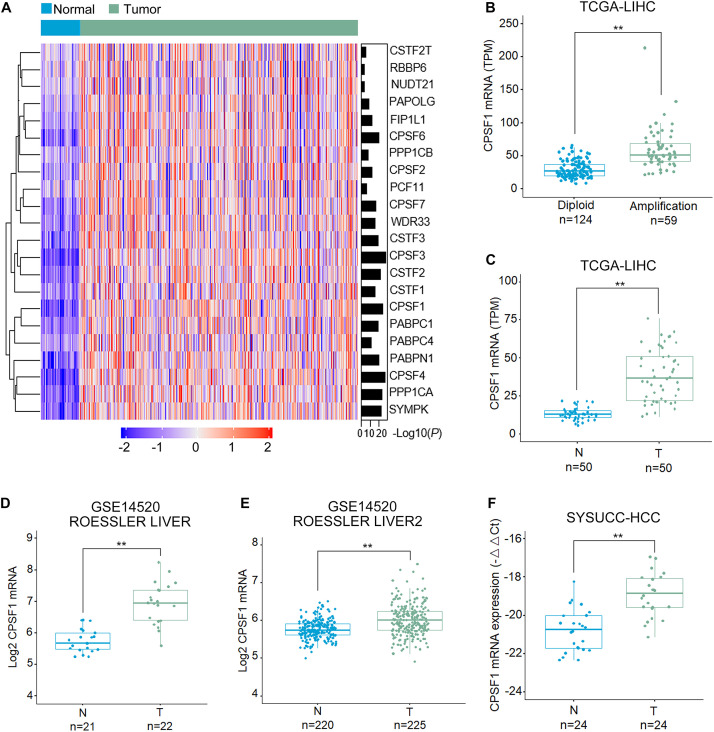
CPSF1 expression is increased in HCC tissues. **(A)** Heatmap comparison of the mRNA level of genes involved in the CPA complex in TCGA LIHC (liver hepatocellular carcinoma) and normal liver samples. **(B)** Boxplots illustrating the correlation between genome amplification and mRNA level of CPSF1. Boxplots comparing the mRNA expression of CPSF1 in tumor (T) and non-tumor (N) tissue samples in the **(C)** TCGA LIHC dataset, **(D)** ROESSLER LIVER, and **(E)** ROESSLER LIVER2 datasets (GSE14520). **(F)** CPSF1 mRNA levels were validated in 24 paired HCC samples using Sun Yat-sen University Cancer Center (SYSUCC) samples. 18S RNA was used to normalize the relative fold changes. Statistical data were assessed using Student *t*-test and presented as the mean ± SD. **P < 0.01.

First, we examined the genome alterations and expression of 22 major CPA factors that drive APA processing in HCC. As indicated by the HCC patient dataset from The Cancer Genome Atlas (TCGA), CPSF1 mRNA was significantly increased in HCC compared to that in normal liver tissues (Figure 1A). Corresponding genome copy number data indicated that CPSF1 amplification correlated with its mRNA upregulation (Figures 1A,B). In 50 paired HCC tissues from the TCGA database, CPSF1 mRNA was relatively elevated in tumor tissues compared to the paired non-tumor tissues (Figure 1C). To further confirm the elevated mRNA expression of CPSF1 in HCC, we searched the data in other public databases. We found that CPSF1 mRNA expression was significantly increased in tumor tissues compared to that in normal tissues in the GEO Roessler liver dataset (Figures 1D,E) that was validated in 24 paired HCC samples collected at the SYSUCC (Figure 1F).

### High CPSF1 Expression Correlates With Poor Patient Prognosis in HCC

**FIGURE 2:**
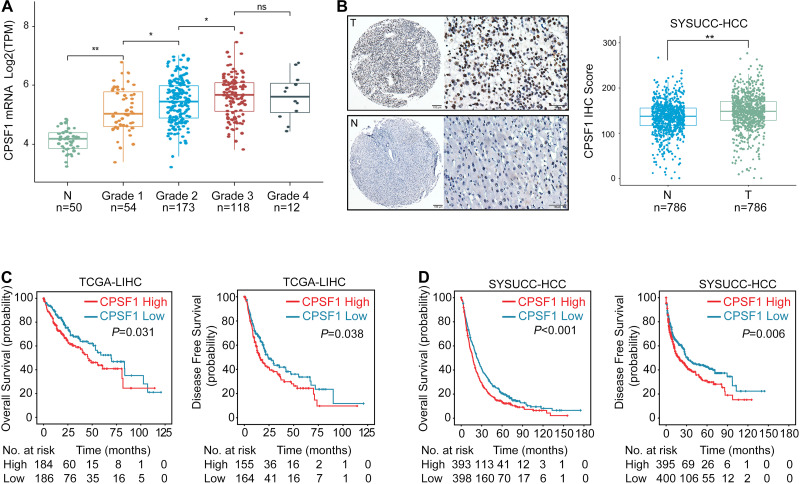
Increased CPSF1 in HCC correlates with unfavorable patient outcome. **(A)** CPSF1 expression was evaluated in TCGA LIHC samples according to different tumor grades. Two-way ANOVA tests showed statistically significant difference between groups (*P* < 2.2e-16). Significant differences between groups were indicated by Student’s *t*-test in Normal vs. Grade 1 (*P* < 0.01), Grade 1 vs. Grade 2 (*P* = 0.011), Grade 2 vs. Grade 3 (*P* = 0.033), but not in Grade 3 vs. Grade 4 (*P* = 0.92). **(B)** Representative images of IHC staining of CPSF1 in tumor (T), and non-tumor (N) tissues. The IHC score for each case is shown on the right. **(C,D)** Correlation between CPSF1 expression and overall survival as well as disease-free survival was determined in the TCGA **(C)**, and SYSUCC cohorts **(D)** by Kaplan–Meier analysis. The high and low CPSF1 groups were separated based on median mRNA level in TCGA and median IHC score in SYSUCC cohorts. *P*-values in **(C)** and **(D)** were calculated by log-rank tests. **P < 0.01 and *P < 0.05.

In terms of its expression and clinical significance, we found that CPSF1 mRNA was elevated from grade 1 to grade 3 (Figure 2A). Next, TMAs containing 786 HCC cases with complete clinicopathological data were evaluated by performing IHC staining for CPSF1 expression (Supplementary Table 2). CPSF1 was primarily localized in the nucleus. Statistical analysis revealed relatively higher expression of CPSF1 in tumor samples than in paired non-tumor samples (Figure 2B).

To identify the correlation between CPSF1 expression and patient outcomes, TCGA data were categorized into high and low groups based on the median CPSF1 mRNA level. Furthermore, the median IHC score of CPSF1 was selected as the cutoff value to group the HCC patients into two groups in the SYSUCC cohort. Survival analyses revealed that CPSF1 overexpression was significantly correlated with worse OS and DFS in TCGA patients (Figure 2C) and SYSUCC cohort (Figure 2D). Furthermore, CPSF1 was statistically associated with poor differentiation and relapse clinicopathological parameters (Supplementary Table 2). Stratified survival analysis revealed that the high CPSF1 group was associated with shorter OS in the larger tumor size (≥5 cm), single or multiple tumors, high AFP levels, poor differentiation, TNM grades, and HBV-positive cases in Supplementary Figure 1. Univariate and multivariate Cox regression analyses indicated that CPSF1 overexpression was an independent predictor of OS (*P* < 0.001) (Supplementary Table 3). Taken together, these results indicate that an increase in CPSF1 expression is a prognostic hallmark of HCC.

### Silencing of CPSF1 Inhibits Cell Growth in HCC

**FIGURE 3:**
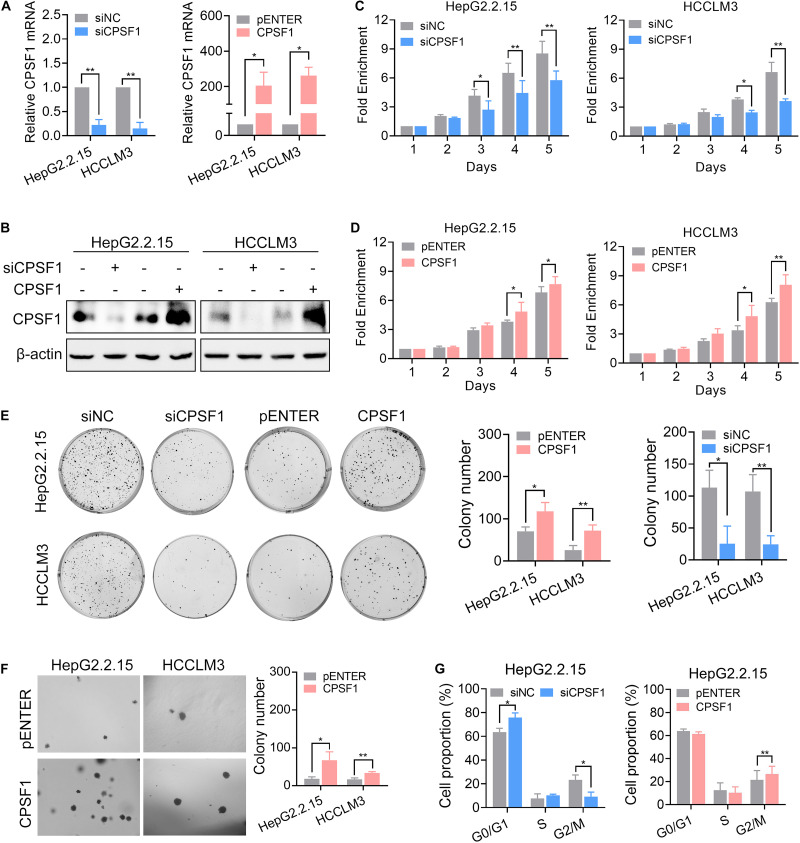
CPSF1-depletion inhibits HCC proliferation and migration. CPSF1 was knocked down or overexpressed in HepG2.2.15 and HCCLM3 cells. The mRNA and protein levels of CPSF1 were determined by qRT-PCR **(A)** and western blot **(B)**, respectively. Statistical significance was calculated by one sample Student’s *t*-test. Cell proliferation in the **(C)** CPSF1-silenced or **(D)** CPSF1-overexpression groups was detected using the CCK8 assay over five consecutive days. The relative absorbance was measured at OD_450_. Fold enrichment was normalized to the absorbance on day 1 and assessed by ANOVA. **(E)** Colony formation assays were used to determine the effect of CPSF1 on cell growth in the indicated groups. The number of colonies was counted using ImageJ software (NIH, Bethesda, MD, United States) and analyzed using Student’s *t*-test. **(F)** Anchorage-independent assays showing that overexpression of CPSF1 promotes colony formation in HepG2.2.15 and HCCLM3 cell lines. **(G)** Flow cytometry assays were used to determine the percentage of cells in various phases of the cell cycle following CPSF1 knockdown or overexpression. Statistical data were assessed using two-way ANOVA. Statistical data are presented as mean ± SD. *P < 0.05 and **P < 0.01.

To explore the biological function of CPSF1 in HCC cells, we depleted CPSF1 expression by transfecting two mixed siRNAs into HepG2.2.15 and HCCLM3 cell lines (Figures 3A,B). Compared to the control cells, silencing of CPSF1 decreased cell proliferation and colony formation as revealed by CCK8 and colony formation assays, respectively (Figures 3C,E). In contrast to CPSF1 silencing, transfection of a CPSF1 ectopic-expression vector into HepG2.2.15 and HCCLM3 cells increased cell growth (Figures 3D,E). These results were further supported by anchorage-independent growth assays (Figure 3F). In cell cycle assays, silencing of CPSF1 increased G0/G1 and reduced G2/M cell proportion, whereas CPSF1 overexpression increased the proportion of G2/M cells in HepG2.2.15 cells (Figure 3G). To further demonstrate that CPSF1 can potentially trigger tumorigenesis in a non-transformed setting, we silenced or overexpressed CPSF1 in the normal liver epithelial cell line, QSG7701. The results indicated that overexpression of CPSF1 promoted cell proliferation and colony formation, whereas knockdown of CPSF1 reduced cell growth in QSG-7701 cells (Supplementary Figure 2).

**FIGURE 4:**
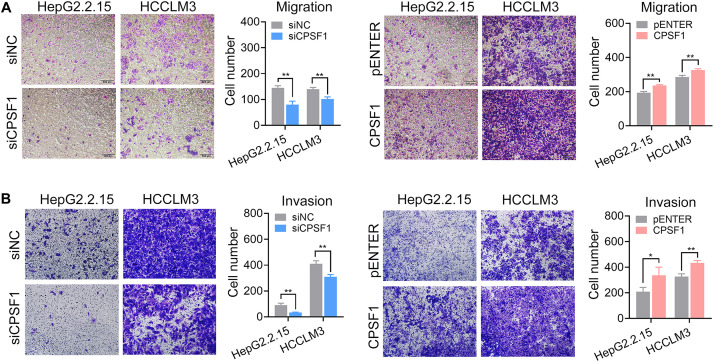
Transwell migration and invasion assays. **(A)** Transwell migration, and **(B)** invasion assays were conducted in the CPSF1 knockdown or CPSF1 overexpression groups to determine the effect of CPSF1 on cell invasion and analyzed using Student’s *t*-test. Statistical data are presented as the mean ± SD. *P < 0.05 and **P < 0.01.

Next, we analyzed the impact of CPSF1 on cell movement. Silencing of CPSF1 inhibited cell migration and invasion, whereas CPSF1 overexpression increased cell migration and invasion, as detected by Transwell assays (Figure 4). These results demonstrated that CPSF1 serves key functions in HCC oncogenesis with a potential role in cell proliferation and metastasis.

### CPSF1 Modulates 3′UTR Alteration in HCC Cells

As CPSF1 is a key cleavage factor responsible for mRNA processing, we examined whether CPSF1 alters polyA site choices, at least in part, leading to changes in 3′ UTR length and RNA transcription. We performed long-read sequencing and constructed a transcriptome.

**FIGURE 5:**
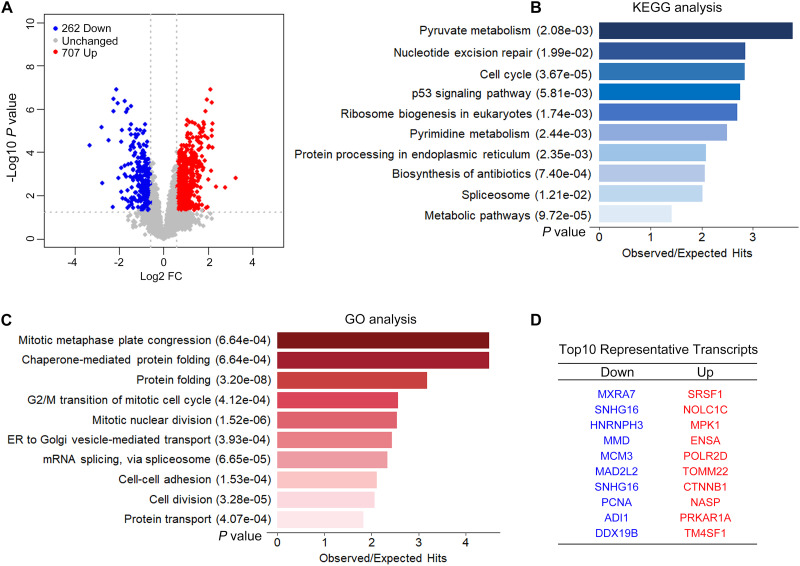
CPSF1 knockdown induced differential expression of transcripts. **(A)** A volcano plot illustrates the expression of transcripts following the silencing CPSF1 in HepG2.2.15 cells. Up- and downregulated transcripts are highlighted as red and blue dots, respectively. Barplots showing the top 10 significantly enriched KEGG pathways **(B)** and GO biological processes **(C)** identified by the gene set analysis using DAVID. **(D)** List of top 10 representative genes down- or upregulated following CPSF1 silencing is presented.

We performed ISO- and RNA-seq in control and CPSF1-siRNA transfected groups. The effect of CPSF1 siRNA transfection was confirmed by RNA-seq data (Supplementary Figure 3). A total of 9,595 transcripts were identified based on the Iso-Seq pipeline analysis, and these transcripts were used to construct the transcriptome. Short reads were mapped to the hg19 reference genome and used to quantify gene expression based on the constructed transcriptome. Knockdown of CPSF1 resulted in 707 upregulated and 262 downregulated transcripts, filtered by a fold change ≥ 1.5, and a *P* < 0.05 (Figure 5A). Gene set overrepresentation analysis identified significantly enriched KEGG pathways related to metabolism, cell cycle, protein processing, and nucleotide excision (Figure 5B) and GO biological processes such as cell division, protein folding, and mRNA splicing (Figure 5C). We performed GO functional enrichment analysis on the up- and downregulated transcripts separately. Results further indicated that the downregulated transcripts were mainly related to cell cycle, whereas the upregulated transcripts were related to RNA processing and protein folding (Supplementary Figure 4). The top 10 representative up- and downregulated transcripts are listed in Figure 5D.

**FIGURE 6:**
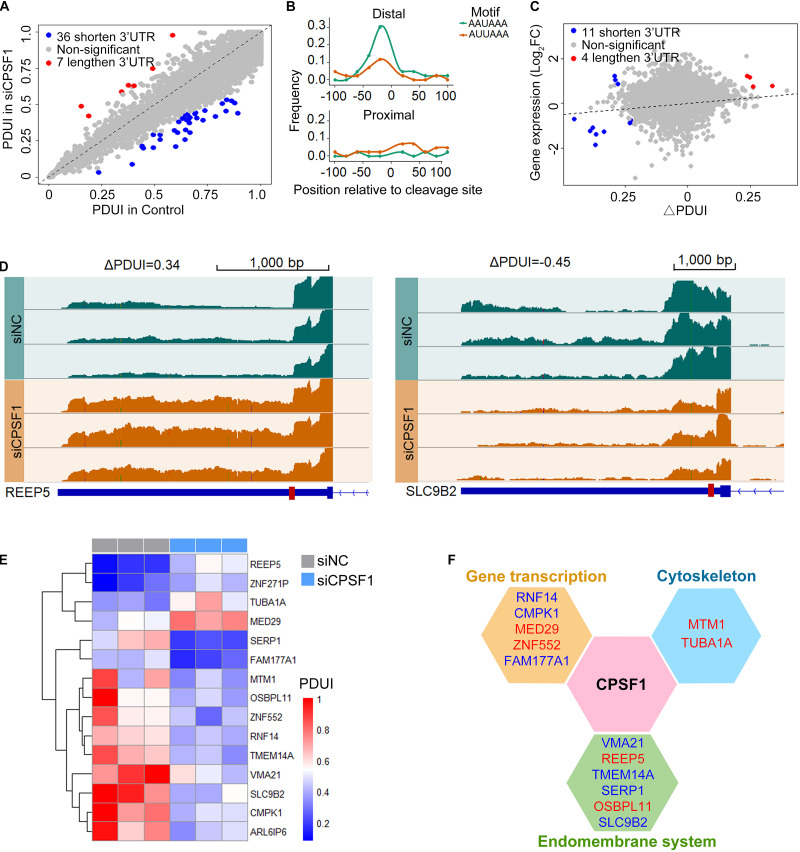
CPSF1 promotes alternative polyadenylation in the 3′UTR. **(A)** Scatterplot showing the percentage of distal polyA site usage index (PDUI) in the control and CPSF1-silenced groups identified by DaPars. Lengthened and shortened transcripts are highlighted with red and blue dots, respectively. **(B)** In the filtered transcripts (differential transcripts), the AAUAAA and AUUAAA polyadenylation signals were highly enriched upstream of the distal compared to the proximal polyadenylation sites following CPSF1 silencing. **(C)** Correlation between PDUI value and gene expression of control and CPSF1-silenced groups. **(D)** Genome browser plots showing the RNA-seq read coverage in representative genes regulated by CPSF1 with 3′ UTR lengthening (REEP5) or shortening (SLC9B2). Red box on the gene body indicates proximal polyadenylation sites predicted by DaPars. **(E)** Heatmap of PDUI showed the clustering of transcripts with both significantly altered mRNA expression and PDUI. **(F)** CPSF1-regulated transcripts are significantly associated with gene transcription, the endomembrane system, and cytoskeleton maintenance. Blue indicates downregulated, whereas red represents upregulated transcripts.

Next, we analyzed the RNA-seq data in the control and CPSF1-siRNA transfected groups using DaPars algorithm to identify 3′ UTR alteration profiles. The significantly differential APA genes were determined by three criteria: *P* < 0.05, ΔPDUI ≥ 0.2, and absolute PDUI fold change ≥ 1.5. Consequently, 36 shortened and seven lengthened transcripts were identified, of which 40 were protein-coding and three were non-coding transcripts (Figure 6A). Interestingly, previous reports indicated that CPSF1 binds to the canonical AAUAAA or similar motifs AUUAAA located 10–50 nt upstream of the 3′ UTR. Analysis of the motif distribution of these 43 transcripts indicated a preferentially high enrichment in the distal polyadenylation sites (Figure 6B), further suggesting that CPSF1 preferentially selects distal compared to proximal polyadenylation sites.

To test the correlation between transcript alteration and APA, we combined the analyses of altered mRNA levels and PDUI (Figure 6C). Upon depletion, we found that 15 transcripts were filtered with both altered mRNA expression and PDUI (Figures 6C–E), including four lengthened (REEP5, ZNF271P, TUBA1A, and MED29) and 11 shortened transcripts. Interestingly, in the 3′ UTR shortened transcripts, three upregulated and eight downregulated transcripts were observed, while 3′ UTR lengthening was upregulated. These included 13 protein-coding and two non-coding transcripts (ZNF271P and ARL6IP6) that were separated into three functions: gene transcription, endomembrane system, and cytoskeleton maintenance (Figure 6F).

To validate these genes related to CPSF1 expression, we separated the TCGA APA events by CPSF1 mRNA median expression score (high vs. low) to compare APAs between patient groups. Analysis of the 15 transcripts regulated by CPSF1 in our RNA-seq data (Figure 6E) revealed four overlapping genes (RNF14, FAM177A1, MED29, and TUBA1A) (Supplementary Figure 5). These results indicate that complex regulatory mechanisms are involved in CPSF1-related gene alteration.

### CPSF1 Modulates Alternative Splicing in HCC Cells

**FIGURE 7:**
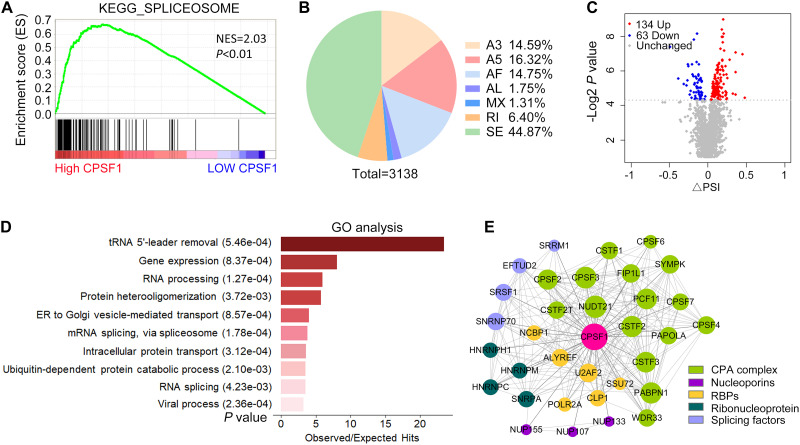
Alternative splicing profiles regulated by CPSF1 in HepG2.2.15 cells. **(A)** GSEA plot showing the correlation between high CPSF1 expression and spliceosome functions. Black vertical lines indicate the gene that correlated with CPSF1 in the gene set. **(B)** Pie chart illustrating the distribution of various types of AS events affected by CPSF1 knockdown as revealed by analysis of RNA-seq data. **(C)** Scatterplot showing the differential AS events identified between the CPSF1-silenced and control groups. **(D)** Barplot listing the top 10 significantly enriched GO biological processes. **(E)** Protein–protein interaction network showing the interaction between CPSF1 and related proteins. The top significant interactions with confidence > 0.95 in the STRING database are included. The size of the nodes and line width indicate the interaction degree and confidence, respectively.

In addition to APA, AS is a pre-mRNA processing event that leads to the diversity and maturation of RNA. Using GSEA of TCGA LIHC data, we found that CPSF1 positively correlated with the function of spliceosome, suggesting a possible role of CPSF1 in AS, in addition to APA (Figure 7A). To test this hypothesis, we further examined the effect of CPSF1 on AS in our RNA-seq data. Surprisingly, a total of 3,138 splicing events of seven types were identified, among which skipping exon (SE) was the most abundant type (Figure 7B and Supplementary Data 1). Next, we filtered the differentially expressed AS events according to *P* < 0.05 and identified 197 significant events that met these criteria (Figure 7C).

Gene Ontology analysis further demonstrated that these AS events were enriched in biological functions related to RNA splicing, viral processing, and protein or vesicle transport (Figure 7D), which was similar to the enrichment results in APA-related genes. Next, we searched the STRING database to identify proteins that were validated to be coexpressed and interact with CPSF1. Interestingly, the top 50 proteins sorted by interaction confidence score included splicing factors (SRSFs), nucleoporins (NUPs), ribonucleoproteins (HNRNPs), and the CPA complex (CPSFs), together with several RBPs assisting the transcription and translation processes (Figure 7E). The network of protein–protein interactions (PPIs) indicated that CPSF1 regulated both AS and APA by cross-interacting with multifunctional proteins that regulate RNA processing, reflecting the connection between AS and APA.

## Discussion

Hepatocellular carcinoma is one of the most common malignancies worldwide. With the development of multi-omics analyses, many differentially expressed genes and signaling networks have been identified. In this study, we report for the first time that CPSF1, the major component of CPA, is upregulated in HCC compared to adjacent non-tumor tissues. The clinical significance of CPSF1 expression is that high expression of CPSF1 indicates unfavorable patient outcome as an independent prognostic factor in HCC. Elevation of CPSF1 mRNA partially accounted for its genomic amplification. Biologically, CPSF1 regulates APA and AS that are correlated with cell proliferation and migration. Thus, our study provides novel insights into the post-transcriptional regulation of HCC growth and suggests that CPSF1 is a potentially novel biomarker for HCC treatment.

The association between APA and tumor progression has been broadly validated in many tumor types, including breast cancer (Fu et al., 2011), colorectal cancer (Morris et al., 2012), glioblastoma tumor (Masamha et al., 2014), leukemia (Lee et al., 2018), and urothelial carcinoma (Chen X. et al., 2018). TCGA datasets also validated that HCC contains many abnormal APA events. However, little attention has been paid to the generation of these APA events during hepatocarcinogenesis. In HCC, the CPA complex, NUDT21 (also known as CPSF5 or CFIm25), has been reported to be a tumor suppressor that inhibits tumor growth and metastasis by regulating APA (Sun et al., 2017; Tan et al., 2018). A zebrafish liver tumor model supported that poorly differentiated tumors had lower expression of liver-specific genes but high expression of genes involved in RNA processing and protein synthesis, including CPSF1 (Lam and Gong, 2006). In the present study, we identified 43 transcripts with altered 3′ UTR following knockdown of CPSF1 in HCC HepG2.2.15 cell line. Among these, 36 shortened and seven lengthened transcripts were identified, indicating that CPSF1 was essential for the selection of distal polyadenylation sites compared to proximal polyadenylation sites.

Interestingly, the shortened transcripts included three upregulated and eight downregulated RNAs, whereas all four lengthened transcripts were upregulated. This suggested that the length of the 3′ UTR dynamically correlates with the expression of the transcripts due to various regulatory mechanisms involved at the mRNA level. For example, changes in the phosphorylation status of RNA polymerase II (RNAPII) affect its elongation rate within the transcription unit and its pausing at the end of 3′ UTR (Tian and Manley, 2017). In addition, the *cis-*elements in the intron can induce longer 3′ ends in histone-globin chimeric genes or enhance polyadenylation rates (Niwa et al., 1990; Pandey et al., 1990). Another reason is that post-transcriptional factors such as microRNAs and accessory RBPs bind to elements located near the regulated polyadenylation sites (Zhu et al., 2007; An et al., 2013). To confirm the hypothesis that microRNAs and RBPs are involved in the regulation of the 3′ UTRs of the CPSF1-altered genes, we analyzed the sequences of the lengthened and shortened transcripts between distal and proximal polyadenylation sites. Using MEME suit analysis, we identified different motifs for lengthened and shortened transcripts. Tomtom analysis revealed that different RBPs and microRNAs bound to this region (Gupta et al., 2007; Supplementary Figure 6A and Supplementary Data 2), supporting motif analysis in the TargetScan database (Supplementary Figure 6B). Among the RBPs predicted by Tomtom analysis mentioned above, we found HNRNPL and HNRNPK that also interact with CPSF1 in the string database (Supplementary Data 3). These results suggested that besides the conserved motifs (AAUAAA or AUUAAA), other motifs within the 3′ UTR transcripts may affect the binding of CPSF1.

Accumulating evidence suggests that AS and APA are not independent processes. The spliceosome and CPA complex are intimately interconnected and functionally intertwined (Martinson, 2011). For example, U1 snRNP, a major component of the spliceosome, protected pre-mRNAs from premature APA splicing. Distinct from its role in splicing, this resulted in differences in mRNA length and isoform expression (Kaida et al., 2010). CPA positions trending to usage of proximal polyadenylation sites with a decrease in U1 level yielded mRNAs with shorter 3′ UTRs and alternatively spliced isoforms (Berg et al., 2012). Several splicing factors have been found to influence polyadenylation (Millevoi et al., 2006). CPSF1 plays a non-canonical role in AS regulation because the CPSF complex usually interacts with the spliceosome (Lutz et al., 1996; Kyburz et al., 2006). Although CPSF1 is the main component of the CPSF complex, it serves AS roles in addition to regulating the APA process. For example, CPSF1 promotes head and neck squamous cell carcinoma growth by regulating AS in cancer-associated genes, such as AKT2, HRAS, TGFBI, and UBE2C (Sakai et al., 2020). CPSF1 causes a splicing switch to inhibit the expression of AR variants and blocks the androgen-independent growth of CRPC cells (Van Etten et al., 2017). CPSF1 regulates the AS of exon 6 in interleukin 7 receptor (IL7R), which is essential for T-cell development and maintenance (Evsyukova et al., 2013). AS cleavage and APA are significant contributors to mRNA transcriptome diversity (Wang et al., 2008; Di Giammartino et al., 2011). These reactions are carried out by the spliceosome and CPA macromolecular complex composed of constant proteins along with the participation of numerous dynamic partners. We chose one representative gene, CMPK1, to validate the RNA-seq results as it was altered in both APA and AS following knockdown of CPSF1 (Supplementary Figure 7). CCK8 results indicated that overexpression of CMPK1 rescued the effect of CPSF1 knockdown on the cellular phenotype (Supplementary Figure 7).

## Conclusion

Our results provide novel insights into the crucial role of the CPA core factor, CPSF1, and its related aberrant APA as well as AS events in HCC. Although further detailed examination is required to understand the exact mechanism underlying CPSF1-mediated cell growth and APA regulation in HCC, our results suggest that CPSF1 serves as an oncogene, and novel prognostic biomarker and potential therapeutic target for patients with HCC.

## Data Availability Statement

The datasets presented in this study can be found in online repositories. The names of the repository/repositories and accession number(s) can be found below: SRA, PRJNA682912.

## Ethics Statement

The studies involving human participants were reviewed and approved by the Institute Research Medical Ethics Committee of Sun Yat-sen University Cancer Center. The patients/participants provided their written informed consent to participate in this study.

## Author Contributions

SC, XW, and JY: conception and design of the study. SC, XY, YH, and MY: *in vitro* experiments. LL and XY: scoring and evaluation of IHC stained slides. ZZ and XW: RNA-seq analysis. SC, XW, and JY: drafting and revision of the manuscript. All authors approved the final version of the manuscript.

## Conflict of Interest

The authors declare that the research was conducted in the absence of any commercial or financial relationships that could be construed as a potential conflict of interest.

## Footnotes

1 https://github.com/PacificBiosciences/Iso-Seq

2 https://github.com/Magdoll/cDNA_Cupcake

3 https://david.ncifcrf.gov

4 http://meme-suite.org/

5 https://string-db.org/

6 http://tc3a.org/

## Supplementary Material

Supplementary Figure 1 Stratified analysis of CPSF1 expression and overall survival in HCC. Stratified analysis of the overall survival of patients with HCC and CPSF1 expression in different subgroups. *P-values* are derived from log-rank tests.

Supplementary Figure 2 CPSF1-depletion inhibits liver cell line proliferation. CPSF1 was silenced by transfection with CPSF1 siRNAs or overexpressed with plasmids in QSG7701 cells. **(A)** CPSF1 protein levels were determined by western blotting. Statistical significance was calculated using Student’s *t*-test. **(B)** Cell proliferation in the CPSF1-silenced or CPSF1-overexpression groups was detected by CCK8 assay over five consecutive days. The relative absorbance was measured at OD_450_. Fold enrichment was normalized to the absorbance on day 1 and assessed using two-way ANOVA. **(C)** Colony formation assays determined the effect of CPSF1 on cell growth. The number of colonies was counted using ImageJ software (NIH, Bethesda, MD, United States) and analyzed using Student’s *t*-test. Statistical data are presented as mean ± SD. **P* < 0.05 and ***P* < 0.01.

Supplementary Figure 3 CPSF1-expression in RNA-seq data. **(A)** Genome browser plots showing the RNA-seq read coverage of CPSF1. **(B)** Gene expression of CPSF1 in the RNA-seq data was analyzed using Student’s *t*-test. Statistical data are presented as mean ± SD. **P* < 0.05.

Supplementary Figure 4 GO enrichment. Barplots showing the enriched GO biological processes identified by the gene set analysis using DAVID in up- (upper) or downregulated transcripts (lower).

Supplementary Figure 5 TCGA APA analysis. TCGA APA events were grouped based on CPSF1 median mRNA expression score (high vs. low) to compare the APA between patient groups. Four representative genes showed statistically significant differences including RNF14, FAM177A1, MED29, and TUBA1A. Statistical analysis was performed using Student’s *t*-test.

Supplementary Figure 6 Motif analysis and interacting RBPs identified in 3′ UTRs regulated by CPSF1. **(A)** Lengthened and shortened sequences of 3′ UTR transcripts regulated by CPSF1 were analyzed using the MEME motif discovery tool, and the significance threshold was set at E-value < 0.05. The motifs were submitted to the Tomtom motif comparison tool for RBP identification. The top three significant RBPs identified are shown. **(B)** Lengthened and shortened sequences of 3′ UTR transcripts regulated by CPSF1 were analyzed by TargetScan tool. The APA event number and microRNA target sites are indicated.

Supplementary Figure 7 CMPK1 was regulated by CPSF1. CPSF1 was knocked down or overexpressed in HepG2.2.15 and HCCLM3 cells. The mRNA level of CMPK1 **(A)** PDUI and **(B)** PSI was determined by qRT-PCR. Statistical significance was calculated by one-sample Student’s *t*-test. **(C)** Cell proliferation was detected using the CCK8 assay over five consecutive days. The relative absorbance was measured at OD_450_. Statistical data are presented as mean ± SD. **P* < 0.05 and ***P* < 0.01.
