# Supplementary figures and images for: Cleavage and Polyadenylation Specific Factor 1 Promotes Tumor Progression via Alternative Polyadenylation and Splicing in Hepatocellular Carcinoma
Source: Front Cell Dev Biol. 2021 Mar 4;9:616835. doi: 10.3389/fcell.2021.616835 (PMC7969726; doi:10.3389/fcell.2021.616835)

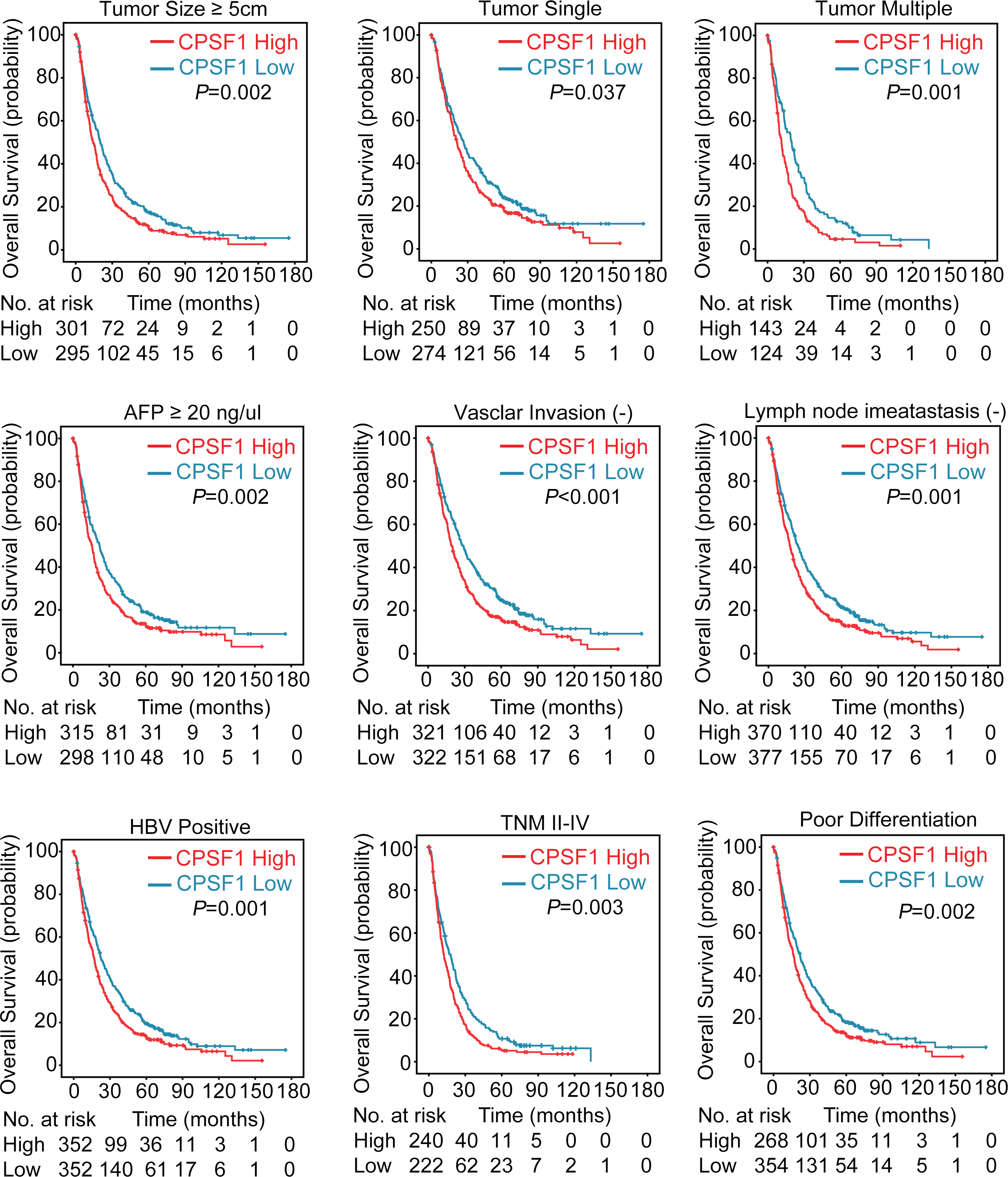

Supplement: Supplementary Figure 1 — Stratified analysis of CPSF1 expression and overall survival in HCC. Stratified analysis of the overall survival of patients with HCC and CPSF1 expression in different subgroups. P-values are derived from log-rank tests. [file Image_1.TIF]

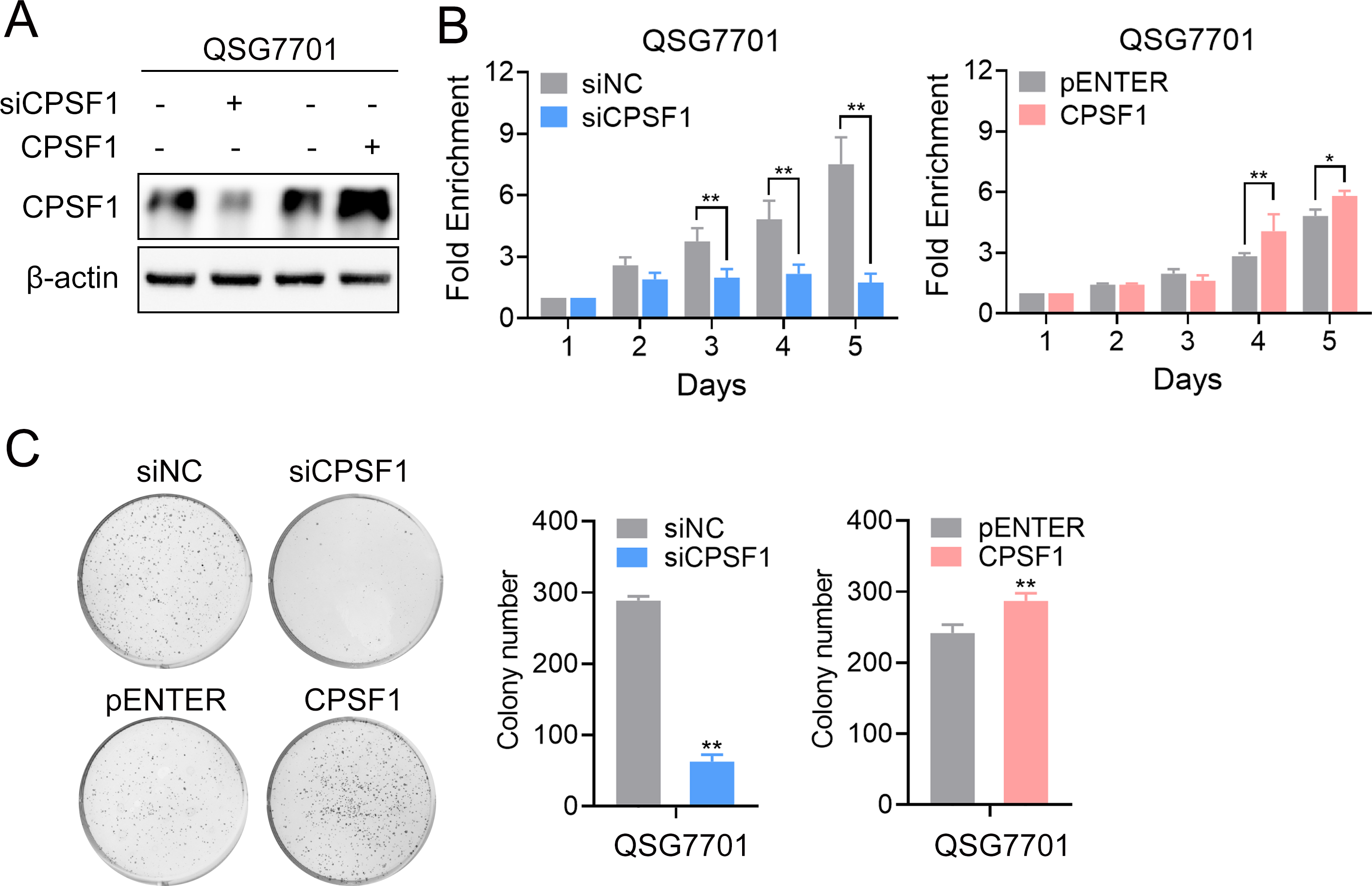

Supplement: Supplementary Figure 2 — CPSF1-depletion inhibits liver cell line proliferation. CPSF1 was silenced by transfection with CPSF1 siRNAs or overexpressed with plasmids in QSG7701 cells. (A) CPSF1 protein levels were determined by western blotting. Statistical significance was calculated using Student’s t-test. (B) Cell proliferation in the CPSF1-silenced or CPSF1-overexpression groups was detected by CCK8 assay over five consecutive days. The relative absorbance was measured at OD450. Fold enrichment was normalized to the absorbance on day 1 and assessed using two-way ANOVA. (C) Colony formation assays determined the effect of CPSF1 on cell growth. The number of colonies was counted using ImageJ software (NIH, Bethesda, MD, United States) and analyzed using Student’s t-test. Statistical data are presented as mean ± SD. *P < 0.05 and **P < 0.01. [file Image_2.TIF]

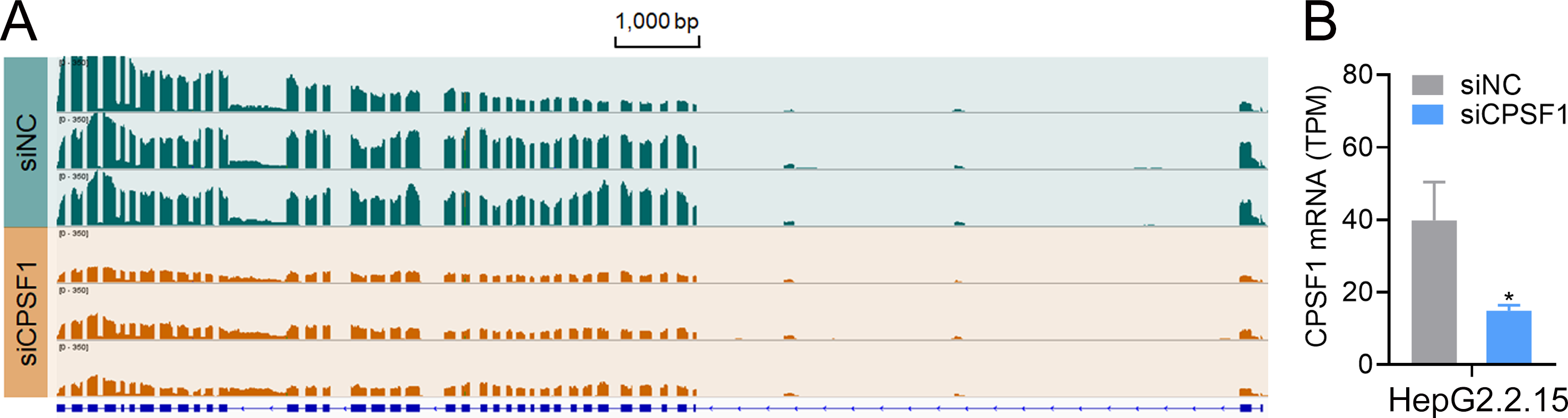

Supplement: Supplementary Figure 3 — CPSF1-expression in RNA-seq data. (A) Genome browser plots showing the RNA-seq read coverage of CPSF1. (B) Gene expression of CPSF1 in the RNA-seq data was analyzed using Student’s t-test. Statistical data are presented as mean ± SD. *P < 0.05. [file Image_3.TIF]

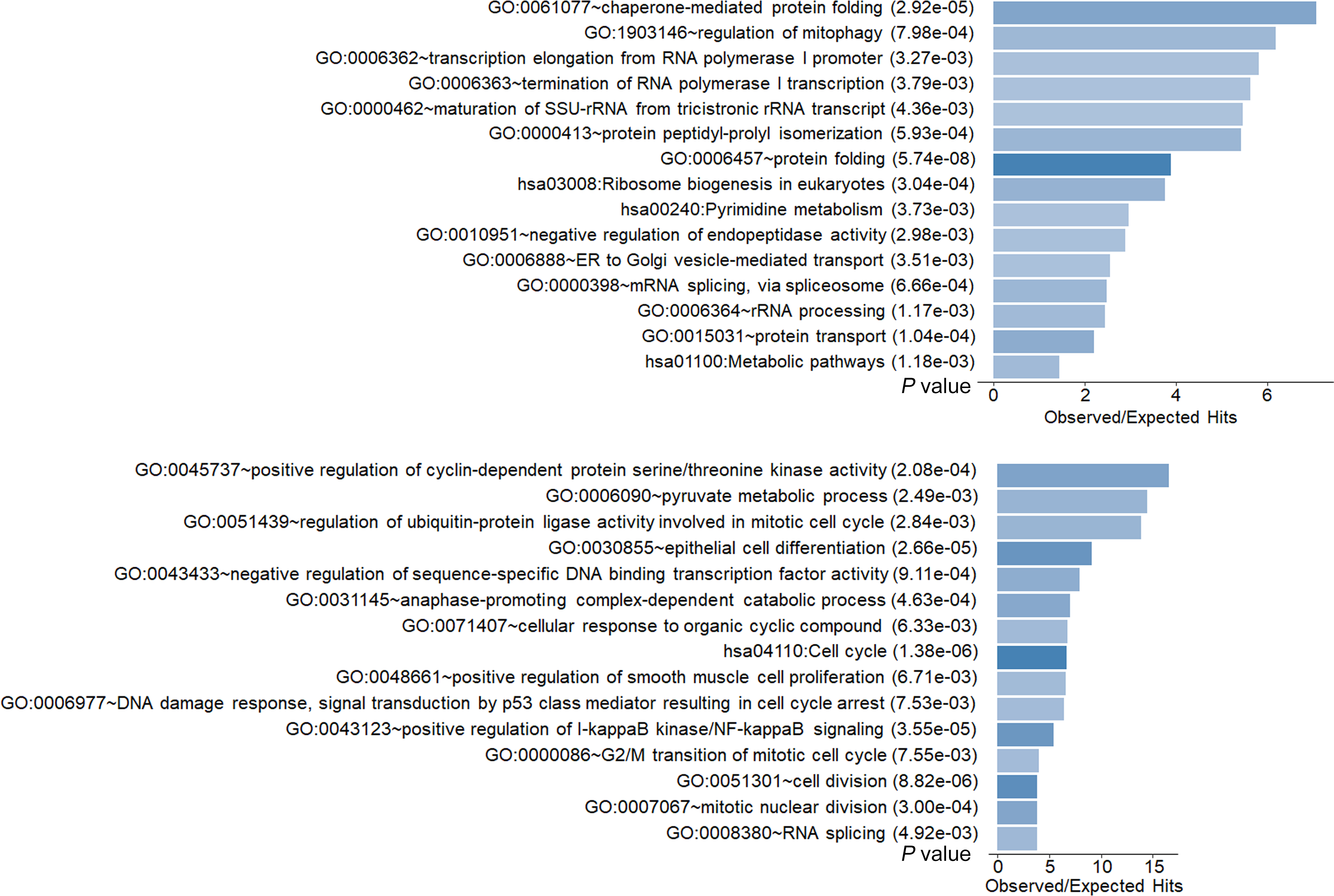

Supplement: Supplementary Figure 4 — GO enrichment. Barplots showing the enriched GO biological processes identified by the gene set analysis using DAVID in up- (upper) or downregulated transcripts (lower). [file Image_4.TIF]

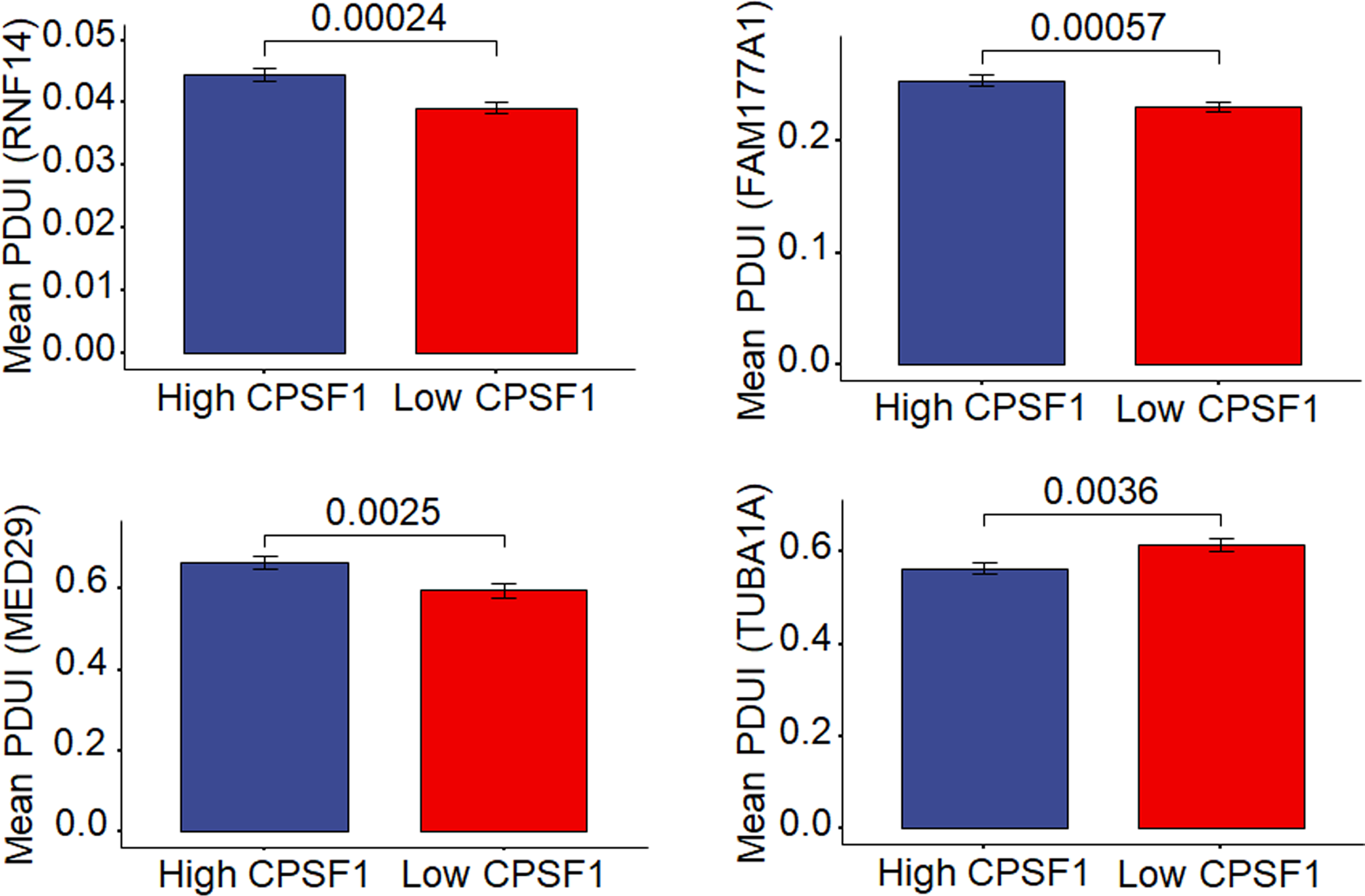

Supplement: Supplementary Figure 5 — TCGA APA analysis. TCGA APA events were grouped based on CPSF1 median mRNA expression score (high vs. low) to compare the APA between patient groups. Four representative genes showed statistically significant differences including RNF14, FAM177A1, MED29, and TUBA1A. Statistical analysis was performed using Student’s t-test. [file Image_5.TIF]

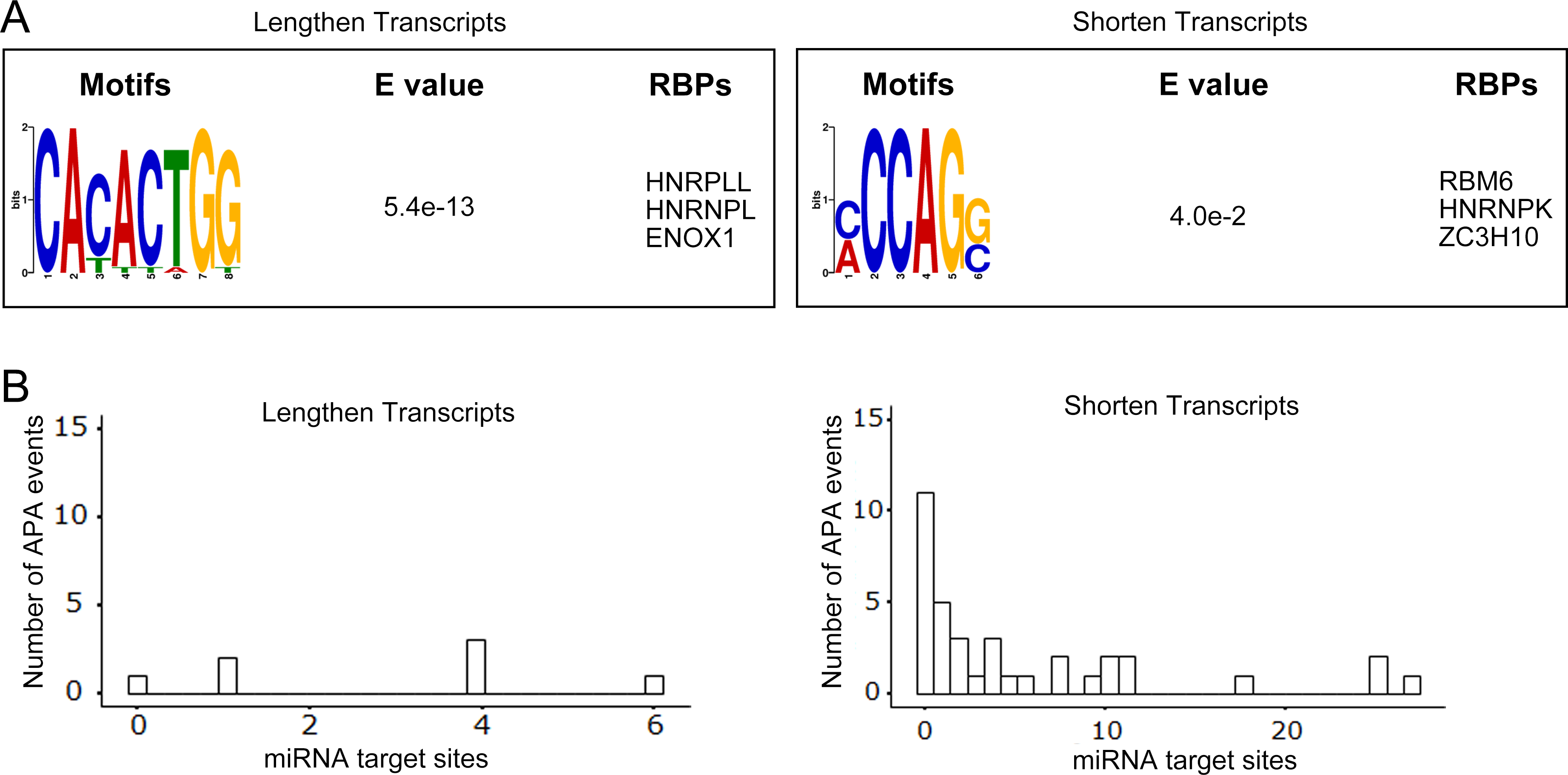

Supplement: Supplementary Figure 6 — Motif analysis and interacting RBPs identified in 3′ UTRs regulated by CPSF1. (A) Lengthened and shortened sequences of 3′ UTR transcripts regulated by CPSF1 were analyzed using the MEME motif discovery tool, and the significance threshold was set at E-value < 0.05. The motifs were submitted to the Tomtom motif comparison tool for RBP identification. The top three significant RBPs identified are shown. (B) Lengthened and shortened sequences of 3′ UTR transcripts regulated by CPSF1 were analyzed by TargetScan tool. The APA event number and microRNA target sites are indicated. [file Image_6.TIF]

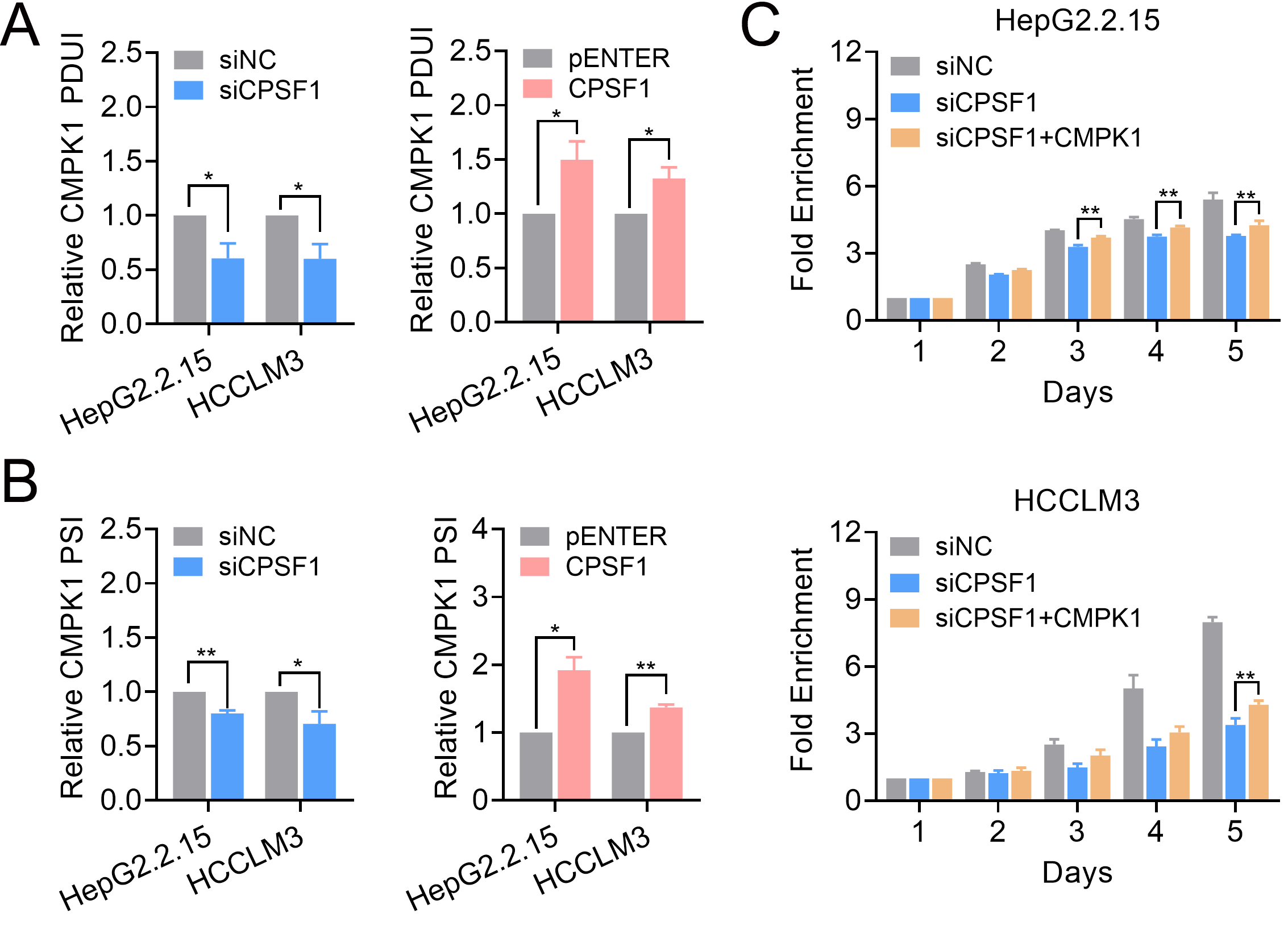

Supplement: Supplementary Figure 7 — CMPK1 was regulated by CPSF1. CPSF1 was knocked down or overexpressed in HepG2.2.15 and HCCLM3 cells. The mRNA level of CMPK1 (A) PDUI and (B) PSI was determined by qRT-PCR. Statistical significance was calculated by one-sample Student’s t-test. (C) Cell proliferation was detected using the CCK8 assay over five consecutive days. The relative absorbance was measured at OD450. Statistical data are presented as mean ± SD. *P < 0.05 and **P < 0.01. [file Image_7.tif]
