# Supplementary material for: Cleavage and Polyadenylation Specific Factor 1 Promotes Tumor Progression via Alternative Polyadenylation and Splicing in Hepatocellular Carcinoma
Source: Front Cell Dev Biol. 2021 Mar 4;9:616835. doi: 10.3389/fcell.2021.616835 (PMC7969726; doi:10.3389/fcell.2021.616835)
Supplement: Supplementary file 11 [file Table_1.DOCX]

**Supplementary Tables**

**Table S1.** siRNAs and Primers

**siRNAs**

| **Identifier Type** | **Sense sequence (5’-3’)** |  |  |  |  |
| --- | --- | --- | --- | --- | --- |
| CPSF1-1 | CUAAGCUUCAAGGAUGCCATT |  |  |  |  |
| CPSF1-2  NC | GCUACUUCGAGGAUAUUUATT  UUCUCCGAACGUGUCACGUTT |  |  |  |  |
| **Primers**   \| **Identifier Type** \| **Sequence (5’-3’)** \| \| --- \| --- \| \| CPSF1-F \| CGCAGCTCTACGTGTACCG \| \| CPSF1-R \| GGACATGACGTTGCCAAAGAA \| \| CMPK1-Total-F \| GGAAGGCAGATGTATCTTTCGTT \| \| CMPK1- Total -R \| TGTTGACTGAAGGTAGGTCTGA \| \| CMPK1-UTR-F \| TGGCATCATGTTGAAGCACC \| \| CMPK1-UTR-R \| TGCCCTATTTGACCCTGTGT \| \| CMPK1-SE-F \| GGAGAGCTGCTTCGTGATGA \| \| CMPK1-SE-R \| ACTGATGGTTATCTCAACTGGTACA \| \| 18S-F \| TGAGAAACGGCTACCACATCC \| \| 18S-R \| ACCAGACTTGCCCTCCAATG \| | | | | |  |

**Table S2.** Correlation of clinicopathological parameters and CPSF1 expression (n=796)

| Variable | CPSF1 expression | | | |
| --- | --- | --- | --- | --- |
|  | All cases | Low expression | High expression | *P* value^a^ |
| Age (years) ^b^ |  |  |  | 0.520 |
| < 49 | 383 | 197 (50.3%) | 186 (49.7%) |  |
| ≥ 49 | 413 | 203 (49.2%) | 210 (50.8%) |  |
| Gender |  |  |  | 0.959 |
| Male | 704 | 354 (50.3%) | 350 (49.7%) |  |
| Female | 92 | 46 (50.0%) | 46 (50.0%) |  |
| HBsAg |  |  |  | 0.691 |
| Positive | 706 | 353 (50.0%) | 353 (50.0%) |  |
| Negative | 90 | 47 (52.2%) | 43 (47.8%) |  |
| AFP (ng/ml) |  |  |  | 0.088 |
| < 20 | 179 | 100 (55.9%) | 79 (44.1%) |  |
| ≥ 20 | 617 | 300 (48.6%) | 317 (51.4%) |  |
| Cirrhosis |  |  |  | 0.851 |
| Yes | 646 | 324 (50.2%) | 322 (49.8%) |  |
| No | 149 | 76 (51.0%) | 73 (49.0%) |  |
| Tumor size (cm) |  |  |  | 0.409 |
| < 5 | 195 | 103 (52.8%) | 92 (47.2%) |  |
| ≥ 5 | 601 | 297 (49.4%) | 304 (50.6%) |  |
| Tumor multiplicity |  |  |  | 0.169 |
| Single | 527 | 274 (52.0%) | 253 (48.0%) |  |
| Multiple | 269 | 126 (46.8%) | 143 (53.2%) |  |
| Differentiation |  |  |  | **0.019** |
| Well | 69 | 44 (63.8%) | 25 (36.2%) |  |
| Poor | 727 | 356 (49.0%) | 371 (51.0%) |  |
| TNM |  |  |  | 0.187 |
| I | 330 | 175 (53.0%) | 155 (47.0%) |  |
| II-IV | 464 | 168 (48.3%) | 171 (51.7%) |  |
| Vascular invasion |  |  |  | 0.780 |
| Yes | 647 | 324 (50.1%) | 323 (49.9%) |  |
| No | 148 | 76 (51.4%) | 72 (48.6%) |  |
| Involucrum |  |  |  | 0.119 |
| Incomplete | 464 | 242 (52.6%) | 220 (47.4%) |  |
| Complete | 332 | 156 (47.0%) | 176 (53.0%) |  |
| LNM |  |  |  | 0.844 |
| No | 750 | 378 (50.4%) | 372 (49.6%) |  |
| Yes | 45 | 22 (48.9%) | 23 (51.1%) |  |
| Relapse |  |  |  | **0.044** |
| Yes  No | 358  437 | 166 (46.4%)  234 (53.5%) | 192 (53.6%)  203 (46.5%) |  |

^a^Chi-square test; ^b^Median age; AFP, alpha-fetoprotein; LNM, lymph node metastasis.

**Table S3.** Univariate and multivariate analyses of clinicopathological and CPSF1 expression for overall survival in overall cohort (n=796).

| Variables | Univariate analysis | |  | | Multivariate analysis | |
| --- | --- | --- | --- | --- | --- | --- |
|  | HR (95% CI) | *P* value | |  | HR (95% CI) | *P* value |
| **Overall survival** |  |  | |  |  |  |
| Age (<49 years) | 0.857 (0.737-0.997) | **0.046** | |  | 0.961 (0.823-1.121) | 0.611 |
| Gender (male) | 0.864 (0.677-1.102) | 0.238 | |  |  |  |
| HBsAg (positive) | 1.153(0.901-1.475) | 0.257 | |  |  |  |
| Liver cirrhosis (yes) | 0.978 (0.803-1.191) | 0.824 | |  |  |  |
| Tumor size (≥5 cm) | 1.627 (1.356-1.953) | **0.000** | |  | 1.423 (1.178-1.718) | **0.000** |
| Tumor multiplicity (multiple) | 1.677 (1.432-1.965) | **0.000** | |  | 1.155 (0.960-1.390) | 0.127 |
| Invonucrum (complete) | 0.726 (0.622-0.848) | **0.000** | |  | 0.839 (0.712-0.988) | **0.036** |
| AFP (≥20 ng/mL) | 1.260 (1.053-1.508) | **0.012** | |  | 1.015 (0.841-1.225) | 0.877 |
| Vascular invasion (yes) | 2.541 (2.103 -3.071) | **0.000** | |  | 1.754 (1.426-2.157) | **0.000** |
| Tumor differentiation (poor) | 1.621 (1.237-2.123) | **0.000** | |  | 1.171 (0.879-1.561) | 0.281 |
| TNM (II-IV) | 2.098 (1.790-2.458) | **0.000** | |  | 1.582 (1.303-1.922) | **0.000** |
| LNM (yes) | 1.877 (1.372-2.569) | **0.000** | |  | 1.423 (1.029-1.967) | **0.033** |
| CPSF1 expression (high) | 1.314 (1.129-1.528) | **0.000** | |  | 1.322 (1.134-1.541) | **0.000** |

AFP, a-fetoprotein; LNM, lymph node metastasis; HR, hazard ratio; CI, confidence interval.
